# Are you into music or sports? Exploring the associations of music and sport identity with mental and physical health through underlying psychological and behavioral pathways

**DOI:** 10.7717/peerj.21286

**Published:** 2026-06-04

**Authors:** Friederike Koehler, Andrew Danso, Alessandro Ansani, Geoff Luck, Suvi Saarikallio

**Affiliations:** Department of Music, Art and Culture Studies, Centre of Excellence in Music, Mind, Body and Brain, University of Jyväskylä, Jyväskylä, Finland

**Keywords:** Leisure, Well-being, Self-efficacy, Health behavior, Physical activity, Musician, Athlete

## Abstract

Leisure activities are essential for building and maintaining a sense of identity, which can significantly impact mental and physical health. Previous research has largely focused on the frequency of engagement in these activities, overlooking the role of identity. Therefore, this cross-sectional online survey examined how music and sports identities relate to health outcomes. To gain more insights into potential internal (psychological) and external (behavioral) pathways underlying these associations, we further explored self-efficacy and health behavior as mediators. A total of 206 adult participants who regularly engage in leisure sports or music activities completed an online survey assessing their identification as (hobby) musicians and sportspersons, mental and physical health, self-efficacy, and health behavior. Using path modeling, we found that a strong sports identity is associated with better mental health, while no direct effects were observed for physical health. Music identity did not directly relate to mental or physical health. However, mediation analyses revealed that both sport and music identities are linked to improved mental health through increased self-efficacy and to better physical health through enhanced health behaviors. These findings suggest that leisure identities play an indirect but crucial role in health, mediated by self-efficacy and health behavior. This knowledge may inform the development of leisure interventions aimed at promoting physical and mental health by raising awareness for the role of leisure identities and addressing self-efficacy beliefs and health behaviors.

## Introduction

How a person spends their free time constitutes a fundamental component of health and well-being (Newman, Tay & Diener, 2014). Leisure activities are commonly defined as voluntary non-work activities that people engage in for pleasure and enjoyment (Elsden et al., 2022). Over recent decades, numerous studies have demonstrated positive associations between leisure participation and both mental and physical health, with some evidence pointing toward potential causal relationships and longer-term health benefits (Fancourt et al., 2021). However, it is not solely the frequency of engagement in a particular leisure activity that matters, but also the extent to which it is integrated into one’s sense of identity. Indeed, identity can significantly predict both behavior and health outcomes (Alfrey et al., 2023). Leisure activities provide individuals with opportunities to construct, affirm, and maintain their identities (Haggard & Williams, 1992).

Despite the prominence of sports and music in people’s leisure-time and their associations with numerous health outcomes (*e.g.*, Koehler et al., 2023b), most prior work has examined these activities separately and focused primarily on frequency of engagement rather than the role of identity (*e.g.*, Elsden et al., 2022). Therefore, with the present study, we aim to explore the mental and physical health differences between identifying as a (hobby) musician *versus* a (hobby) sportsperson. Additionally, we examine underlying psychological and behavioral processes as potential internal (psychological) and external (behavioral) pathways in this association, that is, self-efficacy and health behavior.

### The relationship of music and sports engagement with health

Given the high popularity of sports and musical activities in people’s everyday lives, extensive research on leisure activities has predominantly focused on engagement with sports and music. For instance, Cabane, Hille & Lechner (2016) demonstrate that participation in music and sports activities promotes healthier behaviors, such as reduced smoking rates and overall healthier lifestyles. However, the impact of music and sports engagement might depend on a variety of influencing factors, including activity-specific aspects (*e.g.*, type of sports or musical activity, dosage) and individual differences (*e.g.*, personality traits, beliefs and values, socioeconomic status).

Regarding engagement with sports or leisure-time physical activity in general adult populations, regular physical activity is linked with numerous benefits for physical and mental health (Haskell, Blair & Hill, 2009). Positive associations have been found between sports participation and various dimensions of health-related quality of life, including general health perceptions, vitality, and physical functioning (Wendel-Vos et al., 2004). In adolescent samples, young people who regularly participate in organized sports show better physical fitness than those who do not (see *e.g.*, Kolunsarka et al., 2024). Further, adolescents and young adults who engage in sports leisure activities report higher levels of psychological well-being (Rodríguez-Bravo, De-Juanas & García-Castilla, 2020), with larger effects for those participating in individual sports. Conversely, Murphy, Sweeney & McGrane (2020) found that participation in team sports among adolescents was associated with lower levels of anxiety and depression compared to individual sports, suggesting that the social aspects of team sports provide additional mental health benefits.

Concerning music engagement in leisure-time, much research has focused on music listening, reporting beneficial associations with health (*e.g.*, Linnemann, Strahler & Nater, 2017), although people can engage with music in unhealthy ways leading to maladaptive outcomes as well (Saarikallio, Gold & McFerran, 2015). A recent systematic review and meta-analysis indicates that engaging in active musical activities can have positive effects on both cognitive and psychosocial functioning in adult populations, whereas the benefits of listening to music appear to be mainly confined to the cognitive domain (*e.g.*, Viola et al., 2023). While professional musicians experience various health issues, active amateur musicians report significantly better health compared to both non-musicians and those who are no longer actively playing (Bonde, Juel & Ekholm, 2018). Further, leisure music-making has been associated with greater fulfillment of psychological needs and higher subjective well-being in an experience-sampling study (Koehler & Neubauer, 2020). Music students, compared to an age-matched student population, demonstrate higher well-being but also higher perfectionism and lower stress management, indicating that music engagement can be simultaneously rewarding yet stressful (Araújo et al., 2017). Indeed, recent research advocates a nuanced perspective that also highlights maladaptive engagement with music-making (Koehler et al., 2023a).

### The role of identity in music, sports, and health

As noted earlier, the literature has largely concentrated on participation frequency within both music and sports contexts. In contrast, we turn our attention to leisure identity—specifically, the broader process through which individuals identify as (hobby) musicians or sportspersons. To illustrate, someone might play their guitar once every other month and identify strongly as a hobby musician. In contrast, someone else may sing in a choir every week but has other life domains more pertinent to their identity. Indeed, self- and identity-related processes play crucial roles in healthy human functioning and development (Crocetti et al., 2023). Identity theories commonly depict identity as how we view and define ourselves in a social context (social identity) and as an individual person (personal identity), as well as in our interactions (*e.g.*, identity theory, Alfrey et al., 2023). Leisure activities play a significant role in maintaining and expressing identity, supporting individuals to understand themselves in relation to the world and build a sense of self-consistency (Haggard & Williams, 1992). Adolescents use leisure activities to discover, form, define, and position their identity, and forgo opportunities (Layland, Hill & Nelson, 2018). However, few studies have examined the associations between leisure identity and leisure behavior, let alone health.

In general, engaging in activities such as music or sports can significantly reinforce positive self-identity (Steffens et al., 2021). Regarding music engagement, it has been postulated that due to our innate propensity towards music, every human being has a sense of their own musicality, including identities in music (*i.e.,* self-assessments related to musical aspects) and music in identities (*i.e.,* music as a resource for shaping identity, MacDonald & Saarikallio, 2022). For instance, group music activities can enhance emotional engagement, contributing to a more integrated and positive self-identity (MacRitchie & Garrido, 2019). Correspondingly, identity-related experiences with music affect various aspects of health, including vitality, agency, belonging, and a sense of coherence and meaning (Ruud, 2017). For instance, interviews with metal youth (Rowe & Guerin, 2018) revealed that their metal identities and community protected them from mental health problems. Participating in music and theater can transform identity through experiences of becoming a whole person and exploring multiple identities and perspectives (Ørjasæter et al., 2017).

Identifying as a sportsperson has been associated with both mental and physical health. For instance, Edison, Rizzone & Christino (2021) elucidate both protective and risk factors related to high athletic identity (*i.e.,* the degree of personal connection to sport) in younger populations, noting that while it can protect against burnout, it is also linked to an increased risk of depression following injury. A strong athletic identity has been associated with both higher injury severity and better functional recovery (Renton, Petersen & Kennedy, 2021). Further, multiple sport-related identities include different implications for health. For instance, Miller & Hoffman (2009) report that athletic identity is associated with lower depression scores, whereas the “jock” identity (*i.e.,* strong focus in life on physical prowess and athletic success) correlates with higher odds of suicide attempts. For older adults, the loss of athletic identity, such as during retirement from sports, can negatively impact life satisfaction and mental health (Roberts et al., 2023).

### Underlying psychological and behavioral pathways

Understanding the intricate relationship between identity and health requires delving into the psychological and behavioral pathways that mediate these associations. In this context, psychological pathways refer to the internal processes that influence how individuals perceive and interact with their environment, while behavioral pathways encompass external processes, namely the actions and habits individuals adopt in response to their identities. Exploring these pathways is crucial as they offer insights into how and why leisure identities might have varying impacts on mental and physical health.

One key psychological pathway emerging in health literature is self-efficacy, defined as the belief in one’s own capabilities to organize and execute the courses of action required to manage prospective situations (Bandura, 2000; Bandura, 1993). Unlike task-specific forms, self-efficacy represents a broad sense of personal competence in effectively dealing with a variety of stressful or challenging demands across different domains of functioning (*e.g.*, emotional, social, and cognitive; Luszczynska, Scholz & Schwarzer, 2005). It is expected to improve well-being primarily by regulating affective processes (*e.g.*, reducing anxiety, stress, and depression), promoting coping strategies such as persistence and positive reframing when facing setbacks, as well as supporting resilience through elevated positive cognitive appraisals of challenges (Bandura, 1993; Luszczynska, Scholz & Schwarzer, 2005). Unsurprisingly self-efficacy is considered essential for health behavior engagement (Williams & Rhodes, 2016) and well-being (Parkinson et al., 2023).

Correspondingly, self-efficacy has been investigated as a mediator to predict various health outcomes. For instance, mediation analyses from a longitudinal study (Junker et al., 2019) suggest that self-efficacy plays an essential role in explaining the relationship between social identity and ill health. Similarly, identifying as a (hobby) musician or sportsperson might also have differential effects on self-efficacy and, in turn, affect mental and physical health outcomes. Engaging in these leisure-time activities provides specific tasks and goals to accomplish with immediate feedback, facilitating mastery experiences. Studies show that athletes with high self-efficacy can better cope with stressful events (Guo et al., 2019), while among music students, a lack of self-efficacy was a significant predictor of burnout (Schäfer et al., 2023). Indeed, self-efficacy (considered a key resilience factor) may provide individuals with a stronger sense of control over their environments, potentially buffering against stress and contributing to mental health stability (Schäfer et al., 2023).

Regarding behavioral pathways explaining the link between music and sports identity and mental and physical health, health behavior holds significant potential. Health behavior encompasses actions and routines that contribute to maintaining, restoring, and enhancing health, including smoking, alcohol use, diet, physical activity, sexual behaviors, and physician visits (Conner & Norman, 2017). Numerous studies have shown that increasing physical activity, improving diet, reducing alcohol consumption, quitting smoking, and practicing relaxation techniques can positively affect the management of chronic diseases (Michaelsen & Esch, 2023). A scoping review (Alfrey et al., 2023) highlighted the general role of identity on behavior, suggesting that people prefer to behave in ways that align with their identity. A recent meta-analysis corroborated a positive association between social identity, *i.e.,* the degree to which an individual forms their identity through the groups they belong to, and health behavior (De Hoog & Pat-El, 2024). Accordingly, identifying as a sportsperson might have significant implications for personal values related to health, wellness, and fitness, contributing to greater motivation and adherence to health behaviors, which, in turn, affect mental and physical health. Similarly, musicians might be compliant with their health behaviors to continue engaging in music-making.

### Research objectives

While leisure engagement with music and sports has shown complex but generally beneficial associations with mental and physical outcomes, less research has investigated both activities simultaneously to compare their relative relationships with health, highlighting potential differences. Further, most studies used engagement frequency as a predictor, although the identity of being a (hobby) musician or sportsperson might also play a significant role. Finally, more research is needed on the underlying processes that might explain how these leisure activities can affect health. Against this backdrop, the research questions and hypotheses of this study were:

1. How are music and sports identities associated with mental and physical health?

We hypothesized (*H*_1_) that sports identity would be positively associated with both mental and physical health, with stronger effects expected for sports identity, based on prior literature indicating the benefits of sports participation (*e.g.*, Kolunsarka et al., 2024).

2. Do self-efficacy and health behavior function as parallel mediators in these associations?

We hypothesized (*H*_2*a*_) that both self-efficacy and health behavior would be (positive) mediators in this association. Further, we hypothesized (*H2*_*b*_) that the effects would be stronger for self-efficacy on mental health, and for health behavior (as an immediate behavioral factor) on physical health.

## Methods

### Procedure

The present study comprises a cross-sectional online survey to explore hobby musicians’ and hobby sportspersons’ engagement with music, sports, health, and psychological dispositions. Criteria for inclusion were being hobby musicians or hobby sportspeople (defined as people who engage in leisure music-making or sports at least once a week, without it being their main source of income) and fluency in the English language. The survey collected data on participants’ engagement in music and sports, as well as their subjective health behaviors. Methodological and procedural information is available in a preprint of an earlier version of this study (see *e.g.*, Koehler et al., 2025).

From November 10th, 2023, to March 1st, 2024, a social media recruitment strategy to obtain a sample of hobby musicians and hobby sportspersons was initiated. The strategy used paid, targeted Facebook advertisements, prompting participants to complete an anonymous survey using REDCap (Research Electronic Data Capture, Harris et al., 2009) software. Twenty €25 gift cards were raffled after the survey was closed to incentivize survey completion. According to the Finnish National Board on Research Integrity guidelines (*e.g.*, see pg. 13 in the TENK guidelines: https://www.tenk.fi/sites/tenk.fi/files/ethicalprinciples.pdf and University of Jyväskylä policies, anonymous minimal-risk surveys do not require formal ethical review. The study adhered fully to these guidelines. Participants received a privacy notice in line with GDPR, provided written informed consent prior to participation, and were informed of their rights (*e.g.*, withdrawal, data protection). Participant written informed consent was obtained electronically: participants read the privacy notice and research notification, then confirmed their consent by clicking an “I agree to participate in this study” button on the computer screen before accessing the questionnaire. Furthermore, no direct identifiers were collected, and responses were anonymized at the compilation stage.

### Participants

A sample of 206 participants (*M*_age_: 31.7, *SD* = 15.51, range = 18–82) was recruited from the internet, social media platforms (Facebook and Reddit), and relevant mailing lists: 93 females (45.14%), 108 males (52.42%), and four non-binary individuals (1.94%). The vast majority of the sample was American (54.35%) and British (16.01%) citizens, while the remaining came from 22 different countries. Concerning education, most of the sample (35.95%) declared college/vocational training as the highest educational degree (with 24.27% having completed a bachelor’s degree, 17.96% high school, 12.62% master’s degree, 4.85% doctoral degree, 0.97% middle school). Half of the sample were employees (50.97%), 28.11% were students, and 9.22% were retired workers. The remainder were homemakers (2.42%) and individuals unable to work (2.42%) or out of work (1.45%).

The musical expertise of the sample was quite varied, with 42.23% amateur musicians, 35.52% semi-professional musicians, and 9.22% professional musicians. Typical musical activities included playing in small groups (26.69%), singing in choirs (21.84%), and playing in big bands (11.65%). The largest proportion of hobby musicians (25.33%) played their instrument 2–3 times a week, whereas 24.00% played 4–6 times a week.

In terms of sports expertise, more than half of our sample were amateur sportspeople (56.31%), followed by semi-professionals (20.87%), and professionals (0.48%). Most of them engaged in sports from 1 to 4 times a week (45.62%); another consistent part of the sample exercised 5 times a week or more (21.87%), and an identical part reported 3 times a month or less often.^1^ 1The survey items for music and sports-related activities were displayed based on participants’ responses. The key condition for the survey items to appear is how often they engaged in music or sports activities; specifically, the relative questions appeared if they responded to engaging more than once per week (*N*_music_ = 173 [83.98%]; *N*_sports_ = 160 [77.67%]).

The minimum required sample size to detect mediated effects was determined following the simulation studies by Fritz & MacKinnon (2007). Hypothesizing a partial mediation (*i.e.,* *τ*′ > .39), and α and β paths both halfway between the values for small and medium effect size (*i.e.,* 0.26), 148–196 participants are sufficient to detect mediation with 80% power. Such an effect size was chosen based on prior research showing that most associations in social science research tend to be in the small-to-medium range (*e.g.*, Schäfer & Schwarz, 2019).

### Measures

Physical and mental health were measured through the Physical Functioning and Mental Health subscales of the 36-item Short Form Health Survey (SF-36; Ware & Sherbourne, 1992). In particular, the scoring was executed as per the manual (Ware, 2000). Subsequently, the scores were rescaled to a 1–5 range.

Self-efficacy was assessed using the General Self-Efficacy Scale (GSE-R; Schwarzer & Jerusalem, 1995), while health behavior was measured using the Health Behavior Checklist (HBC; Hampson, Edmonds & Goldberg, 2019). Music and sports identities were each assessed with a single item. Participants responded to “How much do you identify with being a hobby musician?” and “How much do you identify with being a hobby sportsperson?” on a 5-point Likert scale ranging from “not at all” to “very much”. Age was measured as a continuous variable.

### Statistical analysis

The analyses were carried out in the JASP environment (JASP Team, 2024). An assessment of the scales’ reliability was conducted by inspecting Cronbach’s alphas and MacDonald’s omegas prior to the modeling phase. We employed an age-corrected double parallel mediation model to investigate our research questions: Identities were the independent (or exogenous) variables (they were added in the model simultaneously and were not allowed to covary^2^), 2This was done for both theoretical and statistical reasons. In principle, we did not retain such a correlation plausible on an *a priori* theoretical ground, *i.e.,* sport and music belong to two different social domains. Second, we checked their correlation, and we found it to be small in size and not significant, *i.e.,* *ρ* = −0.09, 95% CI [−0.25–0.07], *p* = .182.HBC and SE were the parallel mediators (whose error terms were correlated), and Physical Functioning and Mental Health were the outcome variables. Consistent with recent literature on mediation hypotheses (Zhao, Lynch & Chen, 2010; Memon et al., 2018), the transmittal approach was used to test the mediations. Compared to the classic (Baron & Kenny, 1986) segmentation method, the transmittal approach entails a single hypothesis stating that the mediator (M) mediates the relationship between X and Y without considering hypotheses relating X to M and M to Y (Rungtusanatham, Miller & Boyer, 2014). The indirect effects’ sampling distributions were bootstrapped using a bias-corrected percentile procedure (*N* = 5,000). A statistically significant indirect effect (two-tailed *p* < 0.05) was considered evidence for mediation (Preacher & Hayes, 2004).

In the model, the missing values (*N*_HBC_ = 2, 0.97%; *N*_Gse-R_ = 9, 4.37%; *N*_physical_ = 13, 6.31%; *N*_mental_ = 2, 0.97%; *N*_age_ = 1, 0.48%) were handled using Full Information Maximum Likelihood (FIML) estimation. FIML accounts for all observed data, including missing values, using all available information. Data are assumed to be Missing At Random (MAR). FIML has been shown to be a more reliable estimation method than listwise deletion, pairwise deletion, and mean imputation for missing data (Enders, 2001). Raw data and codebook are available as Supplemental Files. Raw data include all variables used in the analyses, and the codebook provides full definitions and category mappings. The data and analysis scripts are openly available at the Open Science Framework (https://osf.io/nhswb/?view_only=374ce6154a574396b79abb04e3976633).

## Results

### Reliability

Before the modeling phase, the scales’ internal consistency was assessed (see Table 1). All scales were found to be reliable according to common interpretations of acceptability (*i.e.,* α and ω > .70).

### Mediation model

Both music and sport identity showed direct positive effects on the mediating variables, health behavior, and self-efficacy. Specifically, music identity was positively associated with both health behavior (*β* = .16, 95% CI [.03–.29], *SE* = .06, *p* = .011) and self-efficacy (*β* = .18, 95% CI [.01–.35], *SE* = .08, *p* = .028). Notably, the associations of sport identity with health behavior (*β* = .29, 95% CI [.13–.45], *SE* = .08, *p* < .001) and self-efficacy (*β* = .27, 95% CI [.12–.42], *SE* = .07, *p* < .001) were stronger.

Regarding the health outcomes, a direct positive effect of sport identity on mental health was identified, *β* = .21, 95% CI [.06–.34], *SE* = .07, *p* = .005. All other direct effects were non-significant (*p* >.172).

Health behavior significantly mediated the effect of music (*αβ* = .05, 95% CI [.01–.12], *SE* = .02, *p* = .044) and sport identity (*αβ* = .09, 95% CI [.04–.18], *SE* = .03, *p* = .008) on physical health. Similarly, self-efficacy significantly mediated the effect of music (*αβ* = .08, 95% CI [.01–.16], *SE* = .03, *p* = .036) and sport identity (*αβ* = .11, 95% CI [.05–.19], *SE* = .03, *p* = .001) on mental health.

When considering the mediated effects together (*i.e.,* the sum of the paths connecting the identity variables to the health variables *via* the mediators), they were all significant. The strongest connections were those between sport identity and both physical (*αβ* = .12, 95% CI [.05–.20], *SE* = .04, *p* = .002) and mental health (*αβ* = .12, 95% CI [.05–.20], *SE* = .04, *p* = .002). The indirect paths connecting music identity to physical (*αβ* = .07, 95% CI [.02–.14], *SE* = .03, *p* = .016) and mental health (*αβ* = .08, 95% CI [.01–.16], *SE* = .04, *p* = .025) exhibited weaker (but still significant) connections.

Lastly, the overall effects of music identity on physical and mental health (*i.e.,* all direct and mediated paths) were not significant (*p* > .536); whereas the overall effect of sport identity was significant for mental health (*αβ* = .33, 95% CI [.18–.46], *SE* = .07, *p* < .001) but not for physical health (*p* = .491).

**Table 1:** Scale reliabilities.

Scale	Cronbach’s *α*	MacDonald’s *ω*
HBC	.81 [.77,.85]	.82 [.77,.85]
GSE-R	.79 [.74,.83]	.80 [.75,.84]
Physical Health	.95 [.94,.96]	.95 [.94,.96]
Mental Health	.75 [.70,.80]	.77 [.72,.82]

**Notes.**

95% Bootstrapped Confidence Intervals are reported in squared brackets.

HBC Health Behavior Checklist GSE-R General Self-Efficacy Revised

**Figure 1:**
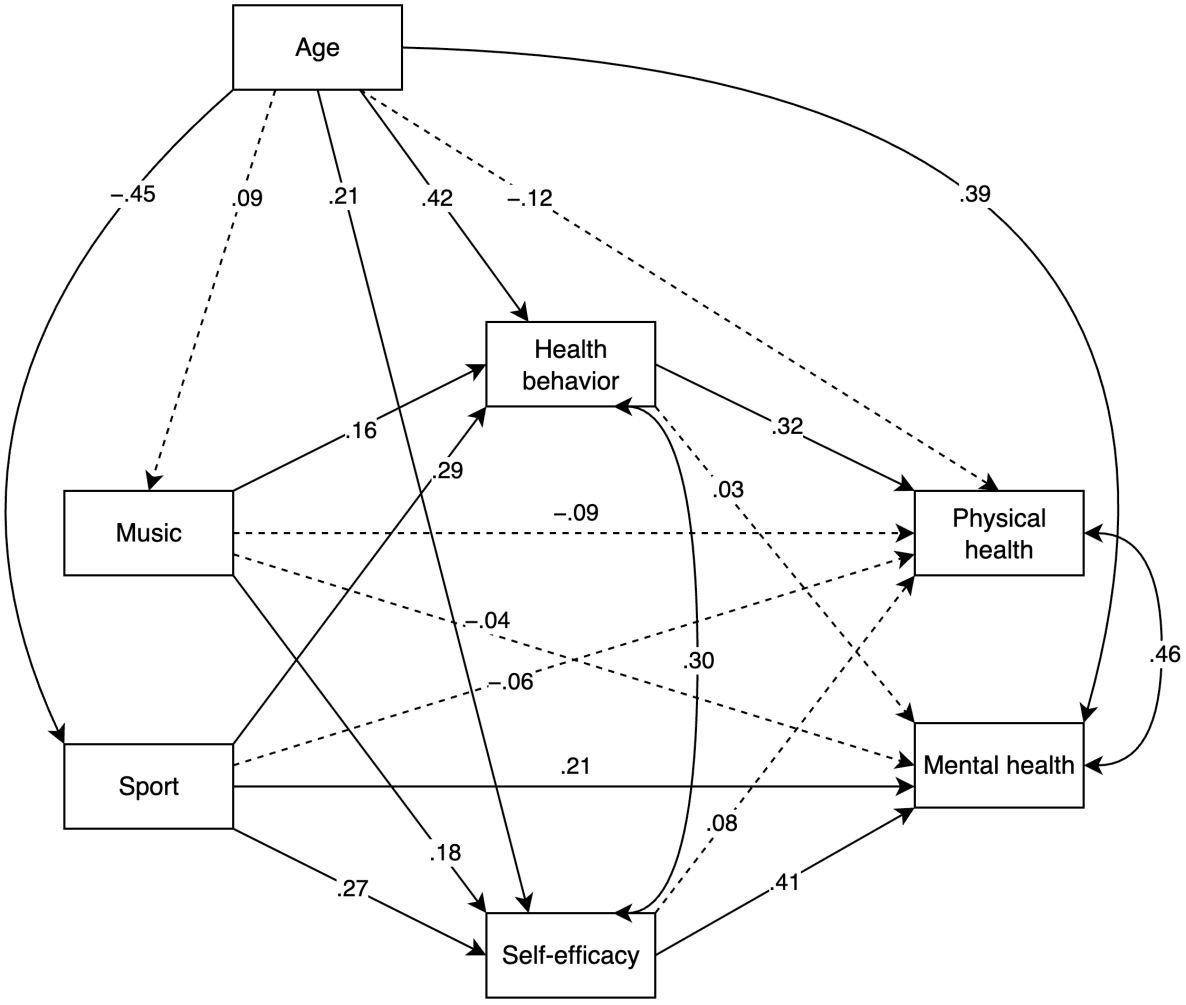
Age-corrected double parallel mediation model. *Note*. The numbers represent standardized coefficients. Continuous lines indicate significant relationships (*p* < .05). Dashed lines indicate non-significant relationships.

Age was significantly related to all variables (see Fig. 1), except for music identity (*p* = .222) and physical health (*p* = .152). A complete list of the estimates can be found in the Appendix.

When checking the residual covariances, mental and physical health covaried significantly (*Cov* = .30, 95% CI [.15–.43], *SE* = .07, *p* < .001). The covariance between the mediator variables was larger (*Cov* = .46, 95% CI [.32–.57], *SE* = .06, *p* < .001).

In sum, stronger identification as a hobby sportsperson was associated with better mental health, both directly and through higher self-efficacy, in this sample. On the contrary, identifying as a hobby musician showed no direct effect on mental health. However, it may associate to improved mental health through self-efficacy. Moreover, neither identity has a direct effect on physical health, but both self-identified hobby musicians and sportspeople improve their physical health by engaging in health behaviors. Both tendencies appear to be more pronounced among hobby sportspeople than among musicians. In statistical terms, the relationships with the mediators are stronger for sportspeople than musicians.

## Discussion

The aim of the present study was to investigate how identifying as a hobby musician or sportsperson is associated with mental and physical health. We hypothesized (*H*_1_) positive associations for both identities, with stronger effects expected for sports identity (*e.g.*, Kolunsarka et al., 2024). We further expected (*H*_2*a*_) that both self-efficacy and health behavior would function as positive mediators in these associations. Specifically, we hypothesized (*H*_2*b*_) that self-efficacy would have stronger indirect effects on mental health, whereas health behavior would have stronger indirect effects on physical health.

### The relationship between sport identity and health

Our analyses indicated a significant positive direct association between sport identity and mental health (*H*_1_). This pattern is consistent with the possibility that identifying as a sportsperson is associated with better mental well-being. Such findings align with prior reports associating athletic identity with lower rates of depression (Miller & Hoffman, 2009) and reduced burnout (Edison, Rizzone & Christino, 2021). Although there is an increased risk of depression among injured sportspeople (Edison, Rizzone & Christino, 2021), and different sport-related identities have differential effects (*e.g.*, the “jock” identity; Miller & Hoffman, 2009), our identity variable specifically solicited identification as a hobby sportsperson. This was intended to capture recreational and leisure-oriented engagement rather than competitive or career-focused athletic identities (*e.g.*, Miller & Hoffman, 2009), which may cautiously describe the positive mental health associations observed here. Identifying as a sportsperson may have implications for mental well-being by fostering a sense of achievement or belonging (*e.g.*, Stevens et al., 2024).

Furthermore, our findings indicate that self-efficacy, rather than health behavior, statistically mediated the association between sport identity and mental health (*H*_2*a*_ and *H*_2*b*_). One plausible explanation is that a stronger identification as a sportsperson may be associated with higher levels of general self-efficacy (*e.g.*, Bandura, 1993). In turn, such generalized efficacy beliefs could plausibly be linked to more favorable mental health through processes such as the regulation of affective states (*e.g.*, attenuated anxiety and depression), greater use of adaptive coping strategies (*e.g.*, persistence and positive reframing when facing setbacks), and more constructive appraisals of challenges (Bandura, 1993; Luszczynska, Scholz & Schwarzer, 2005). Consistent with the literature positioning general self-efficacy as a salutogenic factor buffering against negative influences on health (Cohrdes & Mauz, 2020), identifying as a sportsperson might consolidate a stronger generalized self-concept of personal competence (*i.e.,* the belief that one is generally capable of handling and coping with a broad range of life situations). In turn, such beliefs could plausibly support greater psychological resilience and contribute to an overall sense of well-being. Furthermore, our results are consistent with studies identifying self-efficacy as a resilience factor (Schäfer et al., 2023). Engagement with sports might facilitate experiences of mastery and success (*e.g.*, improving physical fitness or winning a volleyball match), which may potentially strengthen self-efficacy (Kleppang, Steigen & Finbråten, 2023). Athletes with strong self-efficacy are more likely to aim for ambitious goals and remain resilient in the face of challenges (Lochbaum et al., 2023), which might translate to other life domains as well. Interestingly, health behavior did not mediate the association between sports identity and mental health, although sports identity had a direct positive effect on health behavior. This indicates that while sports identity might be associated with increased health behaviors, this does not necessarily lead to better mental health. Health behavior, as defined in our measure (*e.g.*, minimal alcohol consumption, non-smoking, healthy diet), might only indirectly affect mental health in the longer-term, for example, through its effects on physical health (Doan et al., 2023).

Regarding physical health, although our findings did not reveal any direct effects of sports identity, they did provide evidence for mediation *via* health behavior, suggesting that sports identity is positively associated with physical health through increased health behaviors. In other words, individuals with high sports identity engage more in health behaviors and, in turn, are more likely to report better physical health. This finding corroborates the large body of research on the importance of behaviors such as increasing physical activity, improving diet, reducing alcohol consumption, quitting smoking, and practicing relaxation techniques, for physical health (Michaelsen & Esch, 2023). Given that identity reflects an individual’s beliefs and values, those with a strong sports identity may also have a heightened awareness of and value for health, wellness, and fitness, which is reflected in their greater engagement in health-promoting behaviors.

Contrary to our findings on mental health, self-efficacy did not significantly mediate the relationship between sport identity and physical health, indicating that although sport identity might correlate with a firmer belief in one’s ability to handle life situations, this does not necessarily translate into better physical health. Since previous research has consistently shown a positive relationship between self-efficacy and physical health (*e.g.*, Selzler et al., 2020), it may be that the items used as the physical health outcome in our analyses (*i.e.,* how one’s health limits oneself in different everyday activities) do not reflect the impact of self-efficacy on health as measured by other self-report measures or objective assessments of health.

### The relationship between music identity and health

Regarding the association between identifying as a (hobby) musician and mental health, despite no direct effects, our analysis provided evidence for a mediation *via* self-efficacy. This suggests that, through increased self-efficacy, those with a strong music identity might also benefit from better mental health. Building on previous studies that have largely focused on the frequency of leisure music engagement and its positive relationships with well-being (*e.g.*, Koehler & Neubauer, 2020), the finding highlights the importance of music identity in supporting mental health. People report engaging in musical activities mainly to promote identity and agency, which, in turn, can predict well-being (Kiss & Linnell, 2023). Further, our results provide initial insights into underlying psychological processes, suggesting that self-efficacy is one potential pathway. Identifying with being a musician and engaging in music-making might offer individuals opportunities to experience themselves as active agents and to develop skills (*e.g.*, creating sounds on an instrument), to set goals and celebrate achievements (*e.g.*, practicing for an ensemble performance), and thus to foster a positive self-concept. Since music helps cultivate different expressions of self-identity (*e.g.*, Lawendowski & Bieleninik, 2016), a strong music identity may also support self-actualization by enabling one to live one’s interests and passions, laying a foundation for improved self-efficacy and overall well-being. Additionally, identifying as a musician can foster a sense of belonging with like-minded individuals, as one of the most significant roles of music is to promote social bonding (Savage et al., 2021). Active music making offers multiple pathways to strengthen mental well-being, including facilitating connections, enhancing self-development, managing and expressing emotions, and providing respite (Perkins et al., 2020). Similar to our results on sport identity, health behavior significantly mediated the relationship between music identity and physical health (whereas self-efficacy mediated the relationship with mental health).

Regarding physical health, our analysis provided evidence for mediation *via* health behavior, indicating that while a strong music identity might not directly affect physical health, it might do so indirectly by increasing health behaviors. Aligned with our findings on sport identity, the identification as a (hobby) musician might contribute to a motivation to keep active and healthy in life (as reflected in more healthy behavior), since active music engagement can enhance individuals’ sense of purpose and positive outlook in life (Hallam et al., 2014). Findings from a longitudinal study indicate that people with a higher sense of purpose in life also exhibit better health behavior and physical health (Kim et al., 2020). Further, a strong sense of identity as a (hobby) musician might reflect greater use of music in health-related activities (*e.g.*, music listening during exercise) and, in turn, greater compliance with health behaviors (Danso et al., 2025). Music, in this context, plausibly serves as an active ingredient, activating mechanisms of action that influence health behavior (Warran, Burton & Fancourt, 2022; Danso et al., 2025; Danso et al., 2026), while also theoretically eliciting physiological processes that improve health through stress reduction (Taruffi & Koelsch, 2023).

## Limitations

One limitation of this study is its cross-sectional design, which limits our ability to infer causal relationships from the mediation analysis. While the mediation model may suggest potential pathways, a single measurement point makes it challenging to establish temporal precedence among variables and thus definitively determine the direction of causality (Cole & Maxwell, 2003). Future research employing longitudinal or experimental designs would be necessary to validate the inferences proposed in our results.

Further, we used single items to measure music and sports identity, which may have impaired reliability. Future research could employ validated identity scales, such as the Revised Identity Style Inventory (ISI-5; Berzonsky et al., 2013), to gain more nuanced insights into individual differences in identity-related processes. Additionally, our identity measure might have homogenized the enormous number of different sports (*e.g.*, volleyball, yoga, running, golf) and music activities (*e.g.*, playing the violin in an orchestra, singing in a gospel choir, DJing), and their different implications for identity. For instance, different sports can fulfill different needs (Pomohaci & Sopa, 2018), suggesting that future research examine other influencing factors, such as individual *versus* social activities or serious *versus* non-serious leisure engagement. Moreover, defining an activity as a hobby or leisure activity can be subjective. While the frequency of participation might serve as an initial criterion, other elements, such as commitment and purpose, also play a significant role in how individuals perceive their hobbies. Subsequent work should directly compare, or statistically control for, frequency of engagement alongside identity measures to clarify their relative contributions. Future research may also focus on developing and validating multidimensional leisure scales that consider factors beyond mere frequency.

Lastly, our sample consisted mainly of educated individuals from Western cultures, limiting the generalizability of our findings. In particular, the socioeconomic and educational background could affect health outcomes and access to music and sports leisure activities, potentially contributing to selection bias in this study and confounding the results. Recruitment through targeted social media advertisements further increases the likelihood of self-selection bias. Future research should examine these associations in more socioeconomically and culturally diverse samples, including urban-rural comparisons and stratification by income. Moreover, since people often engage in multiple leisure activities, as seen with the overlap of (hobby) musicians and sportspeople in our sample, it may be worthwhile to compare those who exclusively participate in either music or sports to disentangle the distinct and overlapping correlates.

### Implications

The present findings, while cross-sectional, suggest that sport identity may play a somewhat more prominent role in supporting mental health than music identity in this sample. One plausible interpretation is that identifying strongly as a sportsperson is directly associated with better mental health, over and above the indirect pathways observed for both identities. At the same time, the indirect routes *via* self-efficacy and health behavior suggest the potential value of interventions that help individuals develop an increased sense of identification with their chosen leisure activity. Such efforts may plausibly be supported through repeated experiences of mastery, social belonging, and alignment with personal values (*e.g.*, participating in community programs or health-promotion initiatives). These possibilities remain speculative and clearly require careful examination in future experimental and longitudinal studies.

## Conclusion

In the present study, we investigated the associations between music and sport leisure identity and health, examining self-efficacy and health behaviors as potential underlying internal and external processes. Our findings indicate that individuals who strongly identify as a sportsperson tend to have better mental health, though this does not extend to physical health. In contrast, identifying as a musician does not directly relate to either mental or physical health. However, mediation analyses reveal that both sports and music identities contribute to improved mental health *via* increased self-efficacy and to better physical health through enhanced health behavior. These findings point to a potentially indirect role for leisure identities in health, with self-efficacy and health behaviors emerging as plausible psychological and behavioral pathways in this cross-sectional sample. This knowledge may further inform the development of leisure interventions aimed at promoting physical and mental health by fostering strong identification with leisure activities and by addressing individuals’ self-efficacy beliefs and health behaviors.

##  Supplemental Information

10.7717/peerj.21286/supp-1 Supplemental Information 1 Full mediation analysis results for the structural model linking music and sport identity with health outcomes via health behavior and self-efficacy

10.7717/peerj.21286/supp-2 Supplemental Information 2 Data analysis syntax

10.7717/peerj.21286/supp-3 Supplemental Information 3 Dataset file

10.7717/peerj.21286/supp-4 Supplemental Information 4 STROBE Checklist

10.7717/peerj.21286/supp-5 Supplemental Information 5 Codebook of variable names and categorical mappings

## Additional Information and Declarations

### Data Availability

The following information was supplied regarding data availability: The data is available in the Supplemental Files. The Data and syntax used is available at Open Science Framework (OSF) repository: - https://doi.org/10.17605/OSF.IO/MHBCP
