# Supplementary material for: Are you into music or sports? Exploring the associations of music and sport identity with mental and physical health through underlying psychological and behavioral pathways
Source: PeerJ. 2026 Jun 4;14:e21286. doi: 10.7717/peerj.21286 (PMC13242741; doi:10.7717/peerj.21286)
Supplement: Supplemental Information 1 [file peerj-14-21286-s001.docx]

Mediation Analysis

Table of Contents

[Parameter estimates 1](#_Toc223001762)

[Direct effects 1](#_Toc223001763)

[Indirect effects 2](#_Toc223001764)

[Total effects 2](#_Toc223001765)

[Total indirect effects 2](#_Toc223001766)

[Residual covariances 3](#_Toc223001767)

[Path coefficients 3](#_Toc223001768)

[R-Squared 4](#_Toc223001769)

[Mediation model generated by JASP 4](#_Toc223001770)

# Parameter estimates

| Direct effects | | | | | | | | |
| --- | --- | --- | --- | --- | --- | --- | --- | --- |
|  | | | | | | | 95% Confidence Interval | |
|  |  |  | Std. estimate | Std. error | z-value | p | Lower | Upper |
| Music Identity | → | Mental Health | -0.038 | 0.072 | -0.532 | .595 | -0.174 | 0.105 |
| Sport Identity | → | Mental Health | 0.208 | 0.073 | 2.837 | .005 | 0.061 | 0.346 |
| Music Identity | → | Physical Health | -0.096 | 0.070 | -1.366 | .172 | -0.232 | 0.042 |
| Sport Identity | → | Physical Health | -0.064 | 0.085 | -0.761 | .447 | -0.230 | 0.102 |
|  | | | | | | | | |

| Indirect effects | | | | | | | | | | |
| --- | --- | --- | --- | --- | --- | --- | --- | --- | --- | --- |
|  | | | | | | | | | 95% Confidence Interval | |
|  |  |  |  |  | Std. estimate | Std. error | z-value | p | Lower | Upper |
| Music Identity | → | Health Behavior | → | Mental Health | 0.006 | 0.013 | 0.459 | .646 | -0.016 | 0.040 |
| Music Identity | → | Self-Efficacy | → | Mental Health | 0.077 | 0.037 | 2.101 | .036 | 0.012 | 0.161 |
| Sport Identity | → | Health Behavior | → | Mental Health | 0.011 | 0.022 | 0.485 | .628 | -0.031 | 0.059 |
| Sport Identity | → | Self-Efficacy | → | Mental Health | 0.112 | 0.035 | 3.205 | .001 | 0.052 | 0.189 |
| Music Identity | → | Health Behavior | → | Physical Health | 0.054 | 0.027 | 2.015 | .044 | 0.012 | 0.118 |
| Music Identity | → | Self-Efficacy | → | Physical Health | 0.016 | 0.016 | 1.027 | .304 | -0.006 | 0.059 |
| Sport Identity | → | Health Behavior | → | Physical Health | 0.095 | 0.036 | 2.646 | .008 | 0.037 | 0.177 |
| Sport Identity | → | Self-Efficacy | → | Physical Health | 0.023 | 0.023 | 1.016 | .310 | -0.015 | 0.078 |
|  | | | | | | | | | | |

| Total effects | | | | | | | | |
| --- | --- | --- | --- | --- | --- | --- | --- | --- |
|  | | | | | | | 95% Confidence Interval | |
|  |  |  | Std. estimate | Std. error | z-value | p | Lower | Upper |
| Music Identity | → | Mental Health | 0.045 | 0.072 | 0.618 | .536 | -0.090 | 0.198 |
| Sport Identity | → | Mental Health | 0.330 | 0.073 | 4.546 | < .001 | 0.181 | 0.465 |
| Music Identity | → | Physical Health | -0.026 | 0.070 | -0.374 | .708 | -0.159 | 0.113 |
| Sport Identity | → | Physical Health | 0.054 | 0.079 | 0.688 | .491 | -0.099 | 0.208 |
|  | | | | | | | | |

| Total indirect effects | | | | | | | | |
| --- | --- | --- | --- | --- | --- | --- | --- | --- |
|  | | | | | | | 95% Confidence Interval | |
|  |  |  | Std. estimate | Std. error | z-value | p | Lower | Upper |
| Music Identity | → | Mental Health | 0.083 | 0.037 | 2.240 | .025 | 0.013 | 0.161 |
| Sport Identity | → | Mental Health | 0.122 | 0.039 | 3.134 | .002 | 0.054 | 0.206 |
| Music Identity | → | Physical Health | 0.070 | 0.029 | 2.408 | .016 | 0.021 | 0.139 |
| Sport Identity | → | Physical Health | 0.119 | 0.039 | 3.062 | .002 | 0.052 | 0.202 |
|  | | | | | | | | |

| Residual covariances | | | | | | | | |
| --- | --- | --- | --- | --- | --- | --- | --- | --- |
|  | | | | | | | 95% Confidence Interval | |
|  |  |  | Std. estimate | Std. error | z-value | p | Lower | Upper |
| Health Behavior | ~~ | Self-Efficacy | 0.464 | 0.065 | 7.120 | < .001 | 0.319 | 0.577 |
| Mental Health | ~~ | Physical Health | 0.301 | 0.069 | 4.339 | < .001 | 0.152 | 0.428 |
|  | | | | | | | | |

| Path coefficients | | | | | | | | |
| --- | --- | --- | --- | --- | --- | --- | --- | --- |
|  | | | | | | | 95% Confidence Interval | |
|  |  |  | Std. estimate | Std. error | z-value | p | Lower | Upper |
| Health Behavior | → | Mental Health | 0.036 | 0.072 | 0.507 | .612 | -0.103 | 0.177 |
| Self-Efficacy | → | Mental Health | 0.409 | 0.065 | 6.296 | < .001 | 0.280 | 0.536 |
| Music Identity | → | Mental Health | -0.038 | 0.072 | -0.532 | .595 | -0.174 | 0.105 |
| Sport Identity | → | Mental Health | 0.208 | 0.073 | 2.837 | .005 | 0.061 | 0.346 |
| Health Behavior | → | Physical Health | 0.324 | 0.088 | 3.678 | < .001 | 0.138 | 0.482 |
| Self-Efficacy | → | Physical Health | 0.086 | 0.077 | 1.108 | .268 | -0.067 | 0.237 |
| Music Identity | → | Physical Health | -0.096 | 0.070 | -1.366 | .172 | -0.232 | 0.042 |
| Sport Identity | → | Physical Health | -0.064 | 0.085 | -0.761 | .447 | -0.230 | 0.102 |
| Music Identity | → | Health Behavior | 0.167 | 0.066 | 2.535 | .011 | 0.033 | 0.290 |
| Sport Identity | → | Health Behavior | 0.294 | 0.080 | 3.669 | < .001 | 0.133 | 0.449 |
| Music Identity | → | Self-Efficacy | 0.187 | 0.085 | 2.201 | .028 | 0.016 | 0.354 |
| Sport Identity | → | Self-Efficacy | 0.273 | 0.077 | 3.543 | < .001 | 0.121 | 0.418 |
| age | → | Music Identity | 0.092 | 0.075 | 1.226 | .220 | -0.061 | 0.232 |
| age | → | Sport Identity | -0.449 | 0.063 | -7.073 | < .001 | -0.565 | -0.313 |
| age | → | Health Behavior | 0.426 | 0.077 | 5.549 | < .001 | 0.272 | 0.572 |
| age | → | Self-Efficacy | 0.216 | 0.078 | 2.757 | .006 | 0.066 | 0.370 |
| age | → | Mental Health | 0.395 | 0.067 | 5.938 | < .001 | 0.262 | 0.524 |
| age | → | Physical Health | -0.124 | 0.087 | -1.432 | .152 | -0.300 | 0.036 |
|  | | | | | | | | |

| R-Squared | |
| --- | --- |
|  | R² |
| Mental Health | 0.371 |
| Physical Health | 0.112 |
| Health Behavior | 0.182 |
| Self-Efficacy | 0.098 |
| Music Identity | 0.004 |
| Sport Identity | 0.198 |
|  | |

# Mediation model generated by JASP

# dependent regression
Mental Health ~ b11*Health Behavior + b12*Self-Efficacy + c11*Music Identity + c12*Sport Identity
Physical Health ~ b21*Health Behavior + b22*Self-Efficacy + c21*Music Identity + c22*Sport Identity

# mediator regression
Health Behavior ~ a11*Music Identity + a12*Sport Identity
Self-Efficacy ~ a21*Music Identity + a22*Sport Identity

# confounder adjustment
Music Identity ~ age
Sport Identity ~ age
Health Behavior ~ age
Self-Efficacy ~ age
Mental Health ~ age
Physical Health ~ age

# predictor residual covariance
Music Identity ~~ Sport Identity

# mediator residual covariance
Health Behavior ~~ Self-Efficacy

# dependent residual covariance
Mental Health ~~ Physical Health

# effect decomposition
# y1 ~ x1
ind_x1_m1_y1 := a11*b11
ind_x1_m2_y1 := a21*b12
ind_x1_y1 := ind_x1_m1_y1 + ind_x1_m2_y1
tot_x1_y1 := ind_x1_y1 + c11

# y1 ~ x2
ind_x2_m1_y1 := a12*b11
ind_x2_m2_y1 := a22*b12
ind_x2_y1 := ind_x2_m1_y1 + ind_x2_m2_y1
tot_x2_y1 := ind_x2_y1 + c12

# y2 ~ x1
ind_x1_m1_y2 := a11*b21
ind_x1_m2_y2 := a21*b22
ind_x1_y2 := ind_x1_m1_y2 + ind_x1_m2_y2
tot_x1_y2 := ind_x1_y2 + c21

# y2 ~ x2
ind_x2_m1_y2 := a12*b21
ind_x2_m2_y2 := a22*b22
ind_x2_y2 := ind_x2_m1_y2 + ind_x2_m2_y2
tot_x2_y2 := ind_x2_y2 + c22
