# Supplementary material for: Are you into music or sports? Exploring the associations of music and sport identity with mental and physical health through underlying psychological and behavioral pathways
Source: PeerJ. 2026 Jun 4;14:e21286. doi: 10.7717/peerj.21286 (PMC13242741; doi:10.7717/peerj.21286)
Supplement: Supplemental Information 4 [file peerj-14-21286-s004.docx]

STROBE Statement—checklist of items that should be included in reports of observational studies

|  | Item No. | Recommendation | Page  No. | Relevant text from manuscript |
| --- | --- | --- | --- | --- |
| **Abstract** | 1 | (*a*) Indicate the study’s design with a commonly used term in the title or the abstract | 2 | “This cross-sectional online survey examined how music and sports identities relate to health outcomes.” |
|  |  | (*b*) Provide in the abstract an informative and balanced summary of what was done and what was found | 2 | “Using path modeling, we found that a strong sports identity is associated with better mental health, while no direct effects were observed for physical health. Music identity did not directly relate to mental or physical health. However, mediation analyses revealed that both sport and music identities are linked to improved mental health through increased self-efficacy and to better physical health through enhanced health behaviors.” |
| Introduction | | | |  |
| Background/rationale | 2 | Explain the scientific background and rationale for the investigation being reported | 3 | “The relationship of music and sports engagement with health” |
| Objectives | 3 | State specific objectives, including any prespecified hypotheses | 9 | “1. How are music and sports identities associated with mental and physical health?  We hypothesized that both music and sports identities would be positively associated with mental and physical health. Based on more consistent previous research on the benefits of sports participation (e.g., Kolunsarka et al., 2024), we hypothesized that the effects would be stronger for sports identification.  2. Do self-efficacy and health behavior function as underlying mechanisms in this association?  We hypothesized that both self-efficacy and health behavior would be (positive) mediators in this association. Further, we hypothesized that the effects would be stronger for self-efficacy (as a rather stable psychological trait) on mental health, and for health behavior (as an immediate behavioral factor) on physical health.” |
| Methods | | | |  |
| Study design | 4 | Present key elements of study design early in the paper | 9-10 | “Procedure. The present study consists of a cross-sectional online survey to examine hobby musicians' and hobby sportspersons' engagement with music, sports, health, and psychological dispositions.” |
| Setting | 5 | Describe the setting, locations, and relevant dates, including periods of recruitment, exposure, follow-up, and data collection | 9-10 | “From November 10th, 2023, to March 1st, 2024, a social media recruitment strategy to obtain a sample of hobby musicians and hobby sportspersons was initiated.” |
| Participants | 6 | (*a*) *Cohort study*—Give the eligibility criteria, and the sources and methods of selection of participants. Describe methods of follow-up  *Case-control study*—Give the eligibility criteria, and the sources and methods of case ascertainment and control selection. Give the rationale for the choice of cases and controls  *Cross-sectional study*—Give the eligibility criteria, and the sources and methods of selection of participants | 9 | “An invitation to participate was distributed via social media platforms (Facebook and Reddit) and relevant mailing lists. Criteria for inclusion were hobby musicians or hobby sportspeople (defined as people who engage in leisure music-making or sports at least once a week without it being their main source of income) and fluency in the English language. The survey, lasting approximately 30 minutes, gathered data on participants' engagement in music and sports, as well as subjective health behavior.” |
|  |  | (*b*) *Cohort study*—For matched studies, give matching criteria and number of exposed and unexposed  *Case-control study*—For matched studies, give matching criteria and the number of controls per case |  |  |
| Variables | 7 | Clearly define all outcomes, exposures, predictors, potential confounders, and effect modifiers. Give diagnostic criteria, if applicable | 12 | “Measures” |
| Data sources/ measurement | 8* | For each variable of interest, give sources of data and details of methods of assessment (measurement). Describe comparability of assessment methods if there is more than one group | 12-13 | “Statistical analysis” |
| Bias | 9 | Describe any efforts to address potential sources of bias | 13 | “Reliability” |
| Study size | 10 | Explain how the study size was arrived at | 11 | “Hypothesizing a partial mediation (i.e., τ′ > .39), and α and β paths both halfway between the values for small and medium effect size (i.e., 0.26), 148-196 participants are sufficient to detect mediation with 80% power.” |

Continued on next page

| Quantitative variables | 11 | Explain how quantitative variables were handled in the analyses. If applicable, describe which groupings were chosen and why | 12–13 | “Statistical analyses” |
| --- | --- | --- | --- | --- |
| Statistical methods | 12 | (*a*) Describe all statistical methods, including those used to control for confounding | 12–13 | “Statistical analyses” |
|  |  | (*b*) Describe any methods used to examine subgroups and interactions | 12–13 | “Statistical analyses” |
|  |  | (*c*) Explain how missing data were addressed | 12–13 | “Statistical analyses” |
|  |  | (*d*) *Cohort study*—If applicable, explain how loss to follow-up was addressed  *Case-control study*—If applicable, explain how matching of cases and controls was addressed  *Cross-sectional study*—If applicable, describe analytical methods taking account of sampling strategy |  | N/A |
|  |  | (*e*) Describe any sensitivity analyses | 13 | “Before the modeling phase, internal consistency of the scales was assessed (see Table 1). All scales were found to be reliable according to common interpretations of acceptability (i.e., α and ω > .70).” |
| Results | | | | |
| Participants | 13* | (a) Report numbers of individuals at each stage of study—eg numbers potentially eligible, examined for eligibility, confirmed eligible, included in the study, completing follow-up, and analysed | 10–11 | “Participants” |
|  |  | (b) Give reasons for non-participation at each stage |  | N/A |
|  |  | (c) Consider use of a flow diagram |  | N/A |
| Descriptive data | 14* | (a) Give characteristics of study participants (eg demographic, clinical, social) and information on exposures and potential confounders | 10–11 | “Participants” |
|  |  | (b) Indicate number of participants with missing data for each variable of interest | 12–13 | “Statistical analysis” |
|  |  | (c) *Cohort study*—Summarise follow-up time (eg, average and total amount) |  |  |
| Outcome data | 15* | *Cohort study*—Report numbers of outcome events or summary measures over time |  |  |
|  |  | *Case-control study—*Report numbers in each exposure category, or summary measures of exposure |  |  |
|  |  | *Cross-sectional study—*Report numbers of outcome events or summary measures | 13–14 | “Results” |
| Main results | 16 | (*a*) Give unadjusted estimates and, if applicable, confounder-adjusted estimates and their precision (eg, 95% confidence interval). Make clear which confounders were adjusted for and why they were included | 13–14 | “Results” |
|  |  | (*b*) Report category boundaries when continuous variables were categorized | 13 | “Statistical analysis” |
|  |  | (*c*) If relevant, consider translating estimates of relative risk into absolute risk for a meaningful time period |  | N/A |

Continued on next page

| Other analyses | 17 | Report other analyses done—eg analyses of subgroups and interactions, and sensitivity analyses | 13–14 | “Results” |
| --- | --- | --- | --- | --- |
| Discussion | | | | |
| Key results | 18 | Summarise key results with reference to study objectives | 15 | “Discussion – The relationship between sport identity and health” |
| Limitations | 19 | Discuss limitations of the study, taking into account sources of potential bias or imprecision. Discuss both direction and magnitude of any potential bias | 19 | “Limitations” |
| Interpretation | 20 | Give a cautious overall interpretation of results considering objectives, limitations, multiplicity of analyses, results from similar studies, and other relevant evidence | 19–20 | “Limitations and Conclusion” |
| Generalisability | 21 | Discuss the generalisability (external validity) of the study results | 19 | “One limitation of this study is its cross-sectional design, which limits our ability to infer causal relationships from the mediation analysis. While the mediation model may suggest potential pathways, one measurement point makes it challenging to establish temporal precedence of variables and thus definitively determine the direction of causality (Cole & Maxwell, 2003). Future research employing longitudinal or experimental designs would be necessary to validate the inferences proposed in our results.” |
| Other information | |  | | |
| Funding | 22 | Give the source of funding and the role of the funders for the present study and, if applicable, for the original study on which the present article is based | 1 | “This project has received funding from the Research Council of Finland [346210] and from the European Research Council (ERC) under the European Union’s Horizon Europe research and innovation programme [101045747].” |

*Give information separately for cases and controls in case-control studies and, if applicable, for exposed and unexposed groups in cohort and cross-sectional studies.

**Note:** An Explanation and Elaboration article discusses each checklist item and gives methodological background and published examples of transparent reporting. The STROBE checklist is best used in conjunction with this article (freely available on the Web sites of PLoS Medicine at http://www.plosmedicine.org/, Annals of Internal Medicine at http://www.annals.org/, and Epidemiology at http://www.epidem.com/). Information on the STROBE Initiative is available at www.strobe-statement.org.
